# Pediatric Prediction Model for Low Immunoglobulin G Level Based on Serum Globulin and Illness Status

**DOI:** 10.3389/fimmu.2022.825867

**Published:** 2022-02-21

**Authors:** Narissara Suratannon, Phimphika Tantithummawong, Cameron Paul Hurst, Yuda Chongpison, Jongkonnee Wongpiyabovorn, P. Martin van Hagen, Willem A. Dik, Pantipa Chatchatee

**Affiliations:** ^1^ Pediatric Allergy and Clinical Immunology Research Unit, Division of Allergy and Immunology, Department of Pediatrics, Faculty of Medicine, Chulalongkorn University, Bangkok, Thailand; ^2^ King Chulalongkorn Memorial Hospital, The Thai Red Cross Society, Bangkok, Thailand; ^3^ Department of Immunology, Erasmus University Medical Center Rotterdam, Rotterdam, Netherlands; ^4^ Statistics Unit, QIMR Berghofer Medical Research Institute, Herston, QLD, Australia; ^5^ Biostatistics Excellence Center, Research Affairs, Faculty of Medicine, Chulalongkorn University, Bangkok, Thailand; ^6^ Center of Excellence in Immunology and Immune-Mediated Diseases, Department of Microbiology, Faculty of Medicine, Chulalongkorn University, Bangkok, Thailand; ^7^ Department of Internal Medicine, Section Clinical Immunology, Erasmus University Medical Center Rotterdam, Rotterdam, Netherlands; ^8^ Laboratory Medical Immunology, Erasmus University Medical Center Rotterdam, Rotterdam, Netherlands

**Keywords:** globulin, hypogammaglobulinemia, prediction model, screening test, immunoglobulin G

## Abstract

Hypogammaglobulinemia is a condition that requires prompt diagnosis and treatment. Unfortunately, serum immunoglobulin (Ig) measurements are not widely accessible in numerous developing countries. Serum globulin is potentially the best candidate for screening of low IgG level (IgGLo) due to its high availability, low cost, and rapid turnover time. However, multiple factors may influence the probability of prediction. Our study aimed to establish a simple prediction model using serum globulin to predict the likelihood of IgGLo in children. For retrospective data of patients who were suspected of having IgGLo, both serum IgG and globulin were simultaneously collected and measured. Potential factors interfering with serum globulin and IgG levels were investigated for their impact using bivariate binary logistic regression. A multivariate binary logistic regression was used to generate a formula and score to predict IgGLo. We obtained 953 samples from 143 pediatric patients. A strong positive correlation between serum globulin and IgG levels was observed (r=0.83, p < 0.001). A screening test model using serum globulin and illness status was constructed to predict IgGLo. The formula for predicting IgGLo was generated as follows; Predicted score = (2 x globulin (g/dl)) – illness condition score (well=0, sick=1). When the score was <4, the patient has the probability of having IgGLo with a sensitivity of 0.78 (0.71, 0.84), a specificity of 0.71 (0.68, 0.74), PPV of 0.34 (0.29, 0.40) and NPV of 0.94 (0.92, 0.96). This formula will be useful as rapid and inexpensive screening tool for early IgGLo detection, particularly in countries/locations where serum IgG measurement is inaccessible.

## Introduction

Hypogammaglobulinemia refers to a reduction in all types of immunoglobulins (Ig). This condition is strongly associated with recurrent serious infections with encapsulated pyogenic bacteria, including *Streptococcus pneumoniae* and *Haemophilus influenzae type b* (1). IgG is the most abundant idiotype in the circulation. Low IgG level (IgGLo) is defined as a decrease in IgG concentrations at least 2 standard deviations below the mean for the age group (2). Diseases underlying hypogammaglobulinemia and IgGLo may vary from inborn errors of immunity (IEI) (3) to acquired causes (malignancies, severe infections, malnutrition, excessive protein loss from the gastrointestinal tract, skin, and kidneys, or as a side effect of medications) (4, 5). Prompt diagnosis of IgGLo and subsequent treatment with IgG replacement therapy is crucial to prevent long-term morbidity and death (6, 7).

In case of suspected hypogammaglobulinemia, international consensus guidelines recommend prompt measurement of serum IgG (8–10). However, in numerous developing countries, such tests are only available in referral centers or tertiary care hospitals. Therefore, the availability of a simple and inexpensive tool that is widely accessible and applicable would be beneficial, especially for shortening the diagnostic delay of IgGLo. Serum globulin levels, routinely measured in liver function testing, typically yields information on three globulin fractions, namely alpha, beta, and gamma globulins. Serum Igs, in particular IgG, constitutes a significant part of the gamma globulin fraction. Therefore, in developing countries, measurement of serum globulin levels is an attractive candidate to screen for IgGLo due to its high availability, low cost, and rapid turnover time.

A few studies have reported a positive correlation between serum globulin and IgG levels, and demonstrated the feasibility of using serum globulin level as a screening test for hypogammaglobulinemia (8, 9, 11). However, these studies did not consider potential factors that might influence the accuracy of prediction. The main influential factor is serious infection accompanied by an increase in complement proteins, which in turn raises serum globulin levels (9). The simple formula that is suitable for clinical settings has never been established. Also, Data on pediatric populations are limited (12). Moreover, a simple formula that can be applied in clinical settings has never been established. Our study aimed to establish a simple and rapid formula based on inexpensive serum globulin measurement that can be implemented in a clinical setting to predict the probability of IgGLo in patients <18 years of age.

## Material and Methods

### Patients

Medical records from the period of 2011-2021 of patients under 18 years of age (Department of Pediatrics, King Chulalongkorn Memorial Hospital, Bangkok, Thailand) with suspected hypogammaglobulinemia or IgGLo were reviewed. Patients were enrolled in this study when serum IgG and globulin levels measured from the same time point were available. Age, sex, causes of hypogammaglobulinemia, intravenous immunoglobulin (IVIG) replacement, and illness conditions from the time of blood sample collection were retrieved from the medical files. This study was approved by the Ethics Committee of King Chulalongkorn Memorial Hospital, Bangkok, Thailand (IRB No. 504/59).

### Operating Definition

#### Low Serum IgG Levels (IgGLo)

In general, IgGLo is defined as a decrease in IgG concentrations at least 2 standard deviations (SDs) compared to mean age-specific IgG level. However, the risk of recurrent and severe infections generally occurs particularly when serum IgG levels are <500 mg/dl (13). Therefore, in our study, IgGLo was defined as a serum IgG level <500 mg/dl, regardless of age.

#### The Causes of IgGLo

The causes of IgGLo were divided into main two categories: primary and secondary causes. Primary causes of IgGLo related to IEI, particularly B cell differentiation defects. Secondary or acquired causes were assigned to patients when diseases or other extrinsic factors related to IgGLo were identified. These categories are: (1) loss of IgG, including burns, congenital lymphangiectasia, and nephrotic syndrome; (2) drugs that inhibit IgG production or lead to increased IgG metabolization, such as corticosteroids, immune suppressants, and anticonvulsants; (3) malignancies or collagen vascular diseases; (4) viral infections, including Epstein-Barr virus, rubella, HIV, and cytomegalovirus; and (5) other causes related to IgGLo, such as severe malnutrition, severe infections, and prematurity (14).

#### Illness Conditions

Two illness conditions were classified in our study. “Sick” referred to the condition in which the patient had a fever, was admitted to the hospital, or received antibiotics for a treatment at the time blood samples were drawn for IgG and globulin measurement. “Well” referred to the condition in which the individual was in good health when blood samples were drawn.

#### Intravenous Immunoglobulin (IVIG) Replacement Therapy

The half-life of plasma IgG from IVIG is approximately 26–41 days (15). It usually takes 4-5 half-lives to clear the majority of IVIG from the body. IVIG replacement affects vaccination responses for six months after the last dose (16). So in our study, a sample was defined as receiving IVIG replacement when the time of blood sample collection was within six months after the last dose of IVIG.

### Blood Sample Measurement

The globulin fraction (g/dl) was obtained as part of the liver function test, which was determined from the difference between serum total protein and albumin levels. Total protein and albumin levels were measured by the architect biuret method and colorimetric bromocresol green method, respectively. Serum IgG levels (mg/dl) were determined using nephelometry. Both serum IgG and globulin were measured at the same collection time.

### Statistical Analysis

The characteristics of the included patients were described using means and SDs for continuous variables, counts, and percentages for categorical characteristics. Pearson’s correlation was used to measure the strength and direction of the association between serum globulin and IgG levels. Then we formulated the model for predicting IgGLo from serum globulin. Firstly, potential factors that might interfere with serum globulin and IgG levels were selected and investigated using bivariate binary logistic regression. Potential diagnostic factors with a p-value less than 0.2 in the bivariate analysis were then included in a multivariable binary logistic regression and were removed if they were not statistically significant. After identifying the significance of the diagnostic factors and generated models, their utility in diagnosing IgGLo was investigated using receiver operating characteristic (ROC) curves. We fitted the diagnostic model with the variables and removed individual variables to examine whether it reduced model accuracy. Once we decided on the final model, we simplified the model for practical use in clinical situations by rounding the coefficients and comparing the ROC curves of the original and simplified models. We generated the sensitivity, specificity, negative predictive values (NPV) and positive predictive values (PPV), and negative and positive likelihood ratios for the simplified model. Finally, we simplified the cut-off point and investigated whether the diagnostic accuracy of the model was changed. All analyses were conducted using the R statistical package (R core team, 2017); (17, 18), and ROC analysis using the R library pROC (19). Differences were considered statistically significant at p < 0.05.

## Results

### Demographic Data

Demographic data of the patients and samples collected are provided in **Tables 1** and **S1**. In this study, 953 samples from 143 patients were included. Sixty-nine percent of the participants were male. The mean age ± SD of patients having IEI was 7.8 ± 5.1 years old, while the mean age ± SD of patients with acquired IgGLo was 4.76 ± 5.0 years old. Seventy-six percent of the samples were collected from patients with IEIs, half of whom had predominantly antibody deficiencies. The leading cause of acquired IgGLo was sepsis (40.3%). Eighty percent of IEI samples were collected while the patients were in good health, while around sixty percent of samples with acquired IgGLo were collected when the patients were getting sick. Ninety percent of IEI samples and 75 percent of samples with acquired IgGLo were drawn when IVIG was given.

**Table 1:** Demographic Characteristics of Patients and Samples.

Characteristic	Inborn Errors of Immunity (IEI)				Secondary immunodeficiency				Total
	Ab def	Combined	Others	Total subgroup	Sepsis/severe infections	Recurrent pneumonia	Hematologic disorders	Total subgroup	
**Patients (Total numbers = 143)**									
No. of patients; n (%)	16 (11.2)	13 (9.1)	5 (3.5)	34 (23.7)	43 (30.1)	27 (18.9)	39 (27.3)	109 (76.2)	**143 (100)**
Age of the patients; mean years (SD)	6.4 (5.2)	2.9 (4.2)	4.0 (4.1)	4.7 (4.8)	2.8 (4.2)	6.2 (4.1)	4.4 (4.6)	4.2 (4.5)	**4.3 (4.5)**
Male sex; n (%)	11 (44.0)	10 (40.0)	4 (16.0)	25 (73.5)	31 (41.9)	16 (21.6)	27 (36.5)	74 (67.9)	**99 (69.2)**
Patients receiving IVIG; n (%)	15 (93.8)	8 (61.5)	1 (20.0)	24 (79.4)	11 (25.6)	3 (11.1)	1 (2.6)	15 (13.8)	**39 (27.3)**
**Samples (Total numbers = 953)**									
No. of samples; n (%)	411 (43.1)	251 (26.3)	63 (6.6)	725 (76.1)	92 (9.7)	73 (5.2)	63 (6.6)	228 (23.9)	**953 (100)**
Sick condition; n (%)	42 (10.2)	84 (33.5)	8 (12.7)	134 (18.5)	75 (81.5)	42 (57.5)	23 (36.5)	140 (61.4)	**274 (28.8)**
Sample obtained during IVIG administration; n (%)	393 (96.6)	231 (94.7)	53 (100)	677 (93.4)	33 (80.5)	9 (60.0)	3 (23.1)	45 (19.7)	**722 (75.8)**
Serum globulin levels before IVIG administration (g/dl); mean (SD)	1.6 (0.5)	2.4 (0.9)	N/A	2.1 (0.8)	1.5 (0.5)	2.3 (0.8)	2.8 (-)	1.8 (0.7)	**1.9 (0.8)**
Serum globulin levels after IVIG administration (g/dl); mean (SD)	2.2 (0.4)	2.8 (0.9)	2.3 (0.3)	2.4 (0.7)	2.1 (0.7)	2.5 (0.7)	4.2 (0.5)	2.3 (0.9)	**2.4 (0.7)**
Serum IgG levels before IVIG administration (mg/dl); mean (SD)	122.9 (89.5)	453.1 (432.2)	N/A	333.0 (377.1)	324.7 (242.3)	1054.7 (673.0)	1330 (-)	591.0 (528.7)	**467.6 (470.9)**
Serum IgG levels after IVIG administration (g/dl); mean (SD)	686.2 (170.2)	1091.6 (565.0)	693.6 (109.2)	825.1 (404.0)	746.3 (524.2)	823.6 (507.9)	1943.3 (406.7)	841.6 (586.4)	**826.2 (417.1)**

IgG, immunoglobulin G; IVIG, intravenous immunoglobulin; N/A, not available; Ab def, predominantly antibody deficiencies; Combined, immunodeficiencies affecting cellular and humoral immunity; Others, other IEIs including congenital defects of phagocyte and combined immunodeficiencies with associated or syndromic features.

The bold numbers mean for the numbers of the total groups.

### Correlation Between Serum Globulin and IgG Levels

**Figure 1:**
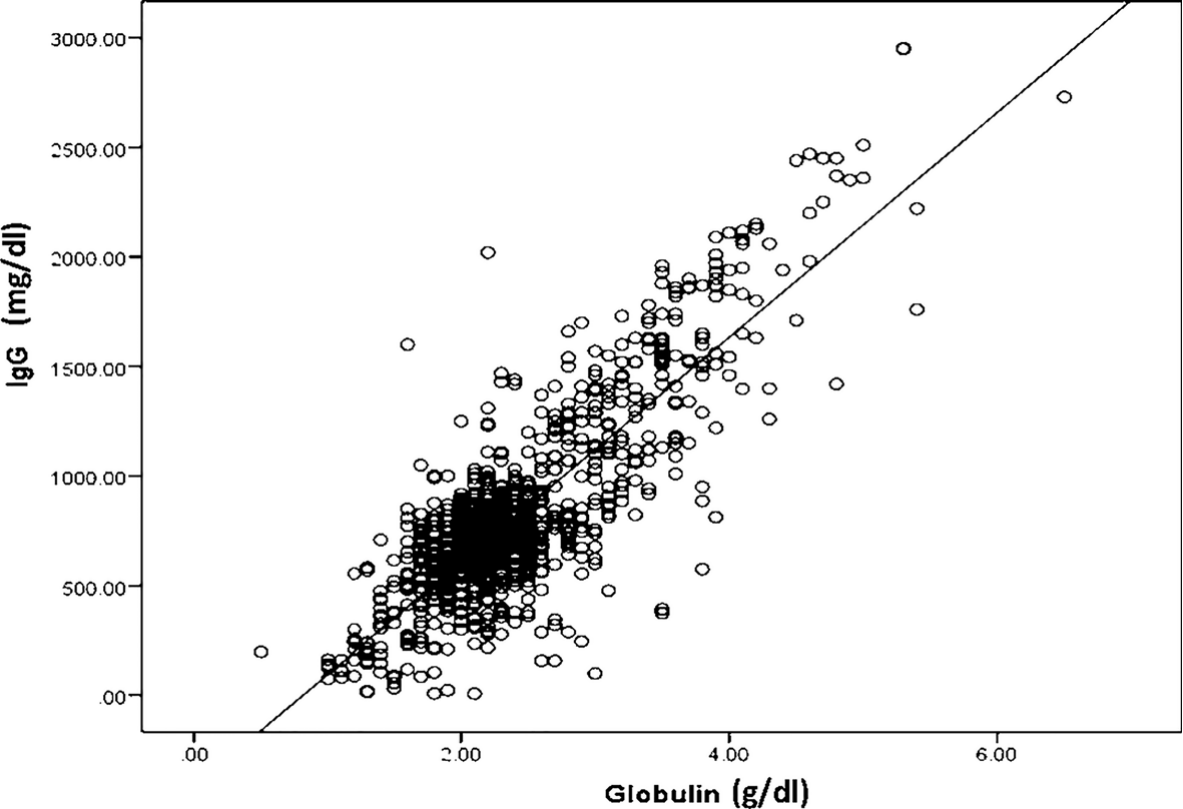
A scatter plot showing a strong positive correlation between serum globulin levels and serum immunoglobulin G levels in all 953 serum samples; r^2 =^ 0.83, p < 0.001.

The association between serum globulin and IgG levels is shown in **Figure 1**. Our results demonstrated a strong positive correlation between serum globulin and IgG levels (r=0.83, p <0.001). Subgroup analysis of 725 samples from patients with IEI and 228 samples from patients with acquired IgGLo also showed a strong correlation between serum globulin and IgG levels (r=0.84, p < 0.001 and r=0.82, p < 0.001, respectively). However, among patients with IEI, patients who had immunodeficiencies affecting cellular and humoral immunity, and other IEIs including congenital defects of phagocyte and combined immunodeficiency (CID) with associated or syndromic features, a stronger correlation was observed (r=0.89, p < 0.001 and r= 0.86, p < 0.001, respectively), while a weaker correlation between serum globulin and IgG levels (r=0.48, p < 0.001) was observed in patients with predominantly antibody deficiencies.

### Prediction Models for IgGLo

Considering that multiple factors can influence serum globulin and IgG levels, we gathered information regarding potential factors including patient age, sex, IVIG replacement, the causes of IgGLo and the illness conditions when serum IgG and globulin levels were determined. We found that patient age, illness conditions, and IVIG replacement were the significant factors that affected serum globulin and IgG levels (p = 0.01, p < 0.001, and p < 0.001 respectively). Using ROC analysis, the area under the curve (AUC) of the model to predict IgGLo taking all significant factors into accounts was 0.8705(0.838, 0.903). When the IVIG replacement was removed, the AUC of the model was 0.8497 (0.815, 0.884). Although the AUC of both models were significantly different (p= 0.0046), the magnitude of difference is quite small (0.02 or 2%). Therefore, IVIG replacement therapy was not included in the final model.

**Figure 2:**
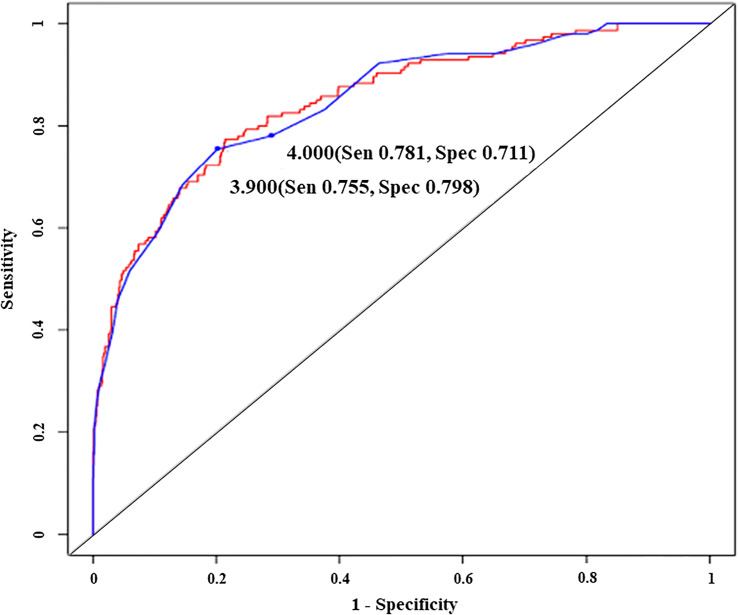
Receiver operating characteristic curves illustrating the diagnostic ability of an original model (red line) and a simplified model with two different cut-off predictive scores (blue line), Sen, sensitivity; Spec, specificity. - Original model; Predictive score = -2.85 x globulin (g/dl) + (1.62 x illness condition score) - Simplified model; Predictive score = 2 x globulin (g/dl) - illness condition score - Illness condition score (well= 0, sick= 1).

Different models from serum globulin, patient age and illness conditions were created and tested for accurate prediction of IgGLo. The models and performance characteristics, including sensitivity, specificity, NPV, and PPV, are provided in **Table 2**. ROC curves illustrate the diagnostic ability of models (**Figure 2**).

**Table 2:** Performance characteristics of the different models for diagnosing low IgG levels.

Model for calculated predictive score (x)	Cutoff score	Sensitivity (95%CI)	Specificity (95%CI)	PPV	NPV
X = age + (60 x globulin) – (25 x I)	127.6	0.77 (0.69, 0.83)	0.79 (0.76, 0.81)	0.41	0.95
X = (-2.85 x globulin) + (1.62 x I)	5.6	0.77 (0.69,0.83)	0.79 (0.76,0.82)	0.42	0.95
X = (2 x globulin) – I	3.9	0.75 (0.68, 0.82)	0.80 (0.77, 0.83)	0.42	0.94
X = (2 x globulin) – I	4.0	0.78 (0.71, 0.84)	0.71 (0.68, 0.74)	0.34	0.94

X, predictive score; I, illness condition score (well= 0, sick= 1); PPV, positive predictive value; NPV, negative predictive value. Age was described in years, the unit of globulin level was g/dl; IgG, immunoglobulin G.

The first prediction model generated was “Predictive score = (- 0.05 x age) + [- 3 x globulin (g/dl) + (1.3 x illness condition score); while well=0, sick=1]” which we further simplified to “Predictive score = age (years) + [60 x globulin (g/dl)] – (25 x illness condition score)”. When the predictive score was ≤ 127.6, the model could predict IgGLo with a sensitivity of 0.77 (0.69, 0.83), specificity of 0.79 (0.76, 0.81), PPV of 0.41 (0.35, 0.47) and NPV of 0.95 (0.93, 0.96). We observed that the removal of “age of patient” from the model did not affect the model performance, however, it provided more simplicity. Therefore, the new model generated without considering patient age was “Predictive score = -2.85 x globulin (g/dl) + (1.62 x illness condition score)”. With the predictive score of ≤ 5.6, the diagnostic performance of the later model was as follows; sensitivity of 0.77 (0.69, 0.83), specificity of 0.79 (0.76, 0.82), PPV of 0.42 (0.36, 0.48) and NPV of 0.95 (0.93, 0.96).

We again rounded up the coefficients of the model to “Predictive score = 2 x globulin (g/dl) - illness condition score”. With a predictive score of ≤ 3.9, the performance of the diagnostic model remained good, with a sensitivity of 0.75 (0.68, 0.82), specificity of 0.80 (0.77, 0.83), PPV of 0.42 (0.36, 0.48) and NPV of 0.94 (0.92, 0.96). Finally, we rounded up the predictive score to the “4” and tested whether the diagnostic value of the model was compromised. Our result showed that with the predictive score ≤ 4, the probability to have IgGLo was not compromised, with a sensitivity of 0.78 (0.71, 0.84), specificity of 0.71 (0.68, 0.74), PPV of 0.34 (0.29, 0.40) and NPV of 0.94 (0.92, 0.96).

## Discussion

Delay in the recognition of IgGLo leads to diagnostic and therapy delay, resulting in devastating consequences. Limited access to IgG measurement is a major reason for delayed diagnosis of this condition in developing countries where measurement of serum IgG levels can be performed only in referral centers. The ultimate goal of this study was to establish a realistic model to predict IgGLo in a clinical setting. We carefully investigated factors potentially influencing IgGLo and generated different models to obtain the most appropriate model and cut-off score to predict IgGLo.

Serum IgG are the major constituents of the serum gamma globulin fraction. Therefore, serum globulin measurement could be an attractive candidate for screening IgGLo. Using serum globulin as a first screening tool is practical because of its high availability, low cost, and rapid turnover time. Previous studies have shown a strong correlation between serum globulin and IgG (8, 9). These studies also proposed serum globulin cut-off levels for predicting IgGLo (8, 9). However, data on children are scarce (12). Importantly, we believe that factors other than serum globulin should also be taken into account for proper prediction of IgGLo. Our results demonstrate that illness condition is an important factor that influences the diagnostic model and performance in children. This is in line with the fact that acute phase proteins are increasing during acute infection and therefore have an impact on the serum globulin level measured under such condition (20). We found no relationship between the causes of IgGLo and the correlation between serum globulin and IgG, making the model more widely applicable.

Even though serum IgG levels in children under one year of age might be influenced by maternal IgG, the age of the patient at the moment of sample collection did not significantly affect our prediction model. In our study 12.3% of samples (117 out of 953 samples) were collected from patients under one year of age (33% of total numbers of patients included). Our analysis revealed that the predictive accuracy of the model was not compromised when the samples obtained at age < 1 year were included. We consider this important since certain patients with IEI with low IgG levels present at very early age (such as severe combined immunodeficiency or X-linked agammaglobulinemia). Therefore, the result of our study is beneficial in early detection of these populations, especially in places where measurement of serum IgG levels is not available.

The reason why the correlation between serum globulin and IgG levels was weaker in patients with predominant antibody deficiencies compared to the other IEI subgroups remains unclear. Additionally, although not proven in our study, some conditions can interfere with the components of serum globulin [e.g. hyperlipoproteinemia (increase alpha-1 globulin), metastatic malignancy (increase alpha-1 and alpha-2 globulin), alpha-1 antitrypsin deficiency (decrease alpha-1 globulin), hemoglobin-haptoglobin complexes secondary to hemolysis (increase alpha-2 globulin), and iron-deficiency anemia with high transferrin (increase beta globulin)] (21). Thus, they may compromise the predictive accuracy for low IgG levels.

In conclusion, early diagnosis of IgGLo is crucial. Serum globulin measurement is a rapid, simple, inexpensive, and widely available tool for predicting IgGLo. We constructed the following screening model/formula for predicting IgGLo in children (age <18 yrs): “Predicted score = (2xglobulin (g/dl)) – illness condition score (well= 0, sick= 1)”, with a score of ≤4 being predictive for IgGLo. We propose that application of this simple and cheap model in developing countries where measurement of serum IgG is generally unavailable would be a valuable tool to reduce diagnostic delay and optimize the use of healthcare resources for the diagnosis of IgGLo in children (age <18 years).

## Data Availability Statement

The original contributions presented in the study are included in the article/**Supplementary Material**. Further inquiries can be directed to pantipa.c@chula.md.

## Ethics Statement

This study was approved by the Ethics Committee of King Chulalongkorn Memorial Hospital, Bangkok, Thailand, IRB No. 504/59. Written informed consent to participate in this study was provided by the participants’ legal guardian/next of kin.

## Author Contributions

NS wrote the first draft of manuscript, collected and analyze data. PT collected data, analyzed the data and wrote the manuscript. CH and YC performed the statistical analysis and wrote the manuscript. JW performed serum globulin and IgG testing and wrote manuscript. PH and WD wrote the manuscript. PC conceptualized of the study and wrote manuscript. All authors contributed to the article and approved the submitted version.

## Funding

This study was supported by Ratchadapiseksompotch Fund, Faculty of Medicine, Chulalongkorn University (RA58/35) and Ratchadaphiseksomphot Endowment Fund Part of the “Research Grant for New Scholar CU Researcher’s Project (RGN_2558_016_08_30), Chulalongkorn University, Bangkok, Thailand.

## Conflict of Interest

The authors declare that the research was conducted in the absence of any commercial or financial relationships that could be construed as a potential conflict of interest.

The handling editor declared a past co-authorship with one of the authors NS.

## Publisher’s Note

All claims expressed in this article are solely those of the authors and do not necessarily represent those of their affiliated organizations, or those of the publisher, the editors and the reviewers. Any product that may be evaluated in this article, or claim that may be made by its manufacturer, is not guaranteed or endorsed by the publisher.

## Abbreviations

Ig, immunoglobulin; IgGLo, low IgG level; IEI, inborn errors of immunity; ESID, European Society for Immunodeficiencies; IVIG, intravenous immunoglobulin; ROC, receiver operating characteristic; NPV, negative predictive values; PPV, positive predictive values; CID, combined immunodeficiency; AUC, area under the curve.
